# No signs of dose escalations of potent opioids prescribed after tibial shaft fractures: a study of Swedish National Registries

**DOI:** 10.1186/1471-2253-14-4

**Published:** 2014-01-13

**Authors:** Zewar Al Dabbagh, Karl-Åke Jansson, Carl-Olav Stiller, Scott Montgomery, Rüdiger J Weiss

**Affiliations:** 1 Department of Molecular Medicine and Surgery, Section of Orthopaedics and Sports Medicine, Karolinska University Hospital, Karolinska Institutet, 171 76 Stockholm, Sweden; 2 Department of Medicine, Clinical Pharmacology Unit, Karolinska University Hospital, Karolinska Institutet, 171 76 Stockholm, Sweden; 3 Department of Medicine, Clinical Epidemiology Unit, Karolinska University Hospital, Karolinska Institutet, 171 76 Stockholm, Sweden; 4 Clinical Epidemiology and Biostatistics, Örebro University Hospital and Örebro University, Örebro, Sweden; 5 Department of Epidemiology and Public Health, University College London, London, UK

**Keywords:** Opioids, Prescriptions, Skeletal trauma, Tibial shaft fracture

## Abstract

**Background:**

The pattern of opioid use after skeletal trauma is a neglected topic in pain medicine. The purpose of this study was to analyse the long-term prescriptions of potent opioids among patients with tibial shaft fractures.

**Methods:**

Data were extracted from the Swedish National Hospital Discharge Register, the National Pharmacy Register, and the Total Population Register, and analysed accordingly. The study period was 2005–2008.

**Results:**

We identified 2,571 patients with isolated tibial shaft fractures. Of these, 639 (25%) collected a prescription for opioids after the fracture. The median follow-up time was 17 (interquartile range [IQR] 7–27) months. Most patients with opioid prescriptions after fracture were male (61%) and the median age was 45 (16–97) years. The leading mechanism of injury was fall on the same level (41%). At 6 and 12 months after fracture, 21% (95% CI 17–24) and 14% (11–17) were still being treated with opioids. Multiple Cox regression-analysis (adjusted for age, sex, type of treatment, and mechanism of injury) revealed that older patients (age >50 years) were more likely to end opioid prescriptions (Hazard ratio 1.5 [95% CI 1.3-1.9]). During follow-up, the frequency of patients on moderate and high doses declined. Comparison of the daily morphine equivalent dose among individuals who both had prescriptions during the first 3 months and the 6th month indicated that the majority of these patients (11/14) did not have dose escalations.

**Conclusions:**

We did not see any signs in registry-data of major dose escalations over time in patients on potent opioids after tibial shaft fractures.

## Background

Previous studies on consumption of opioids in patients with non-cancer pain were either non population-based [1,2], limited to a specific population, such as workers with low back injuries [3-5], or had only a short follow-up [6]. Moreover, studies dealing with concerns of abuse, side effects, and efficacy of long-term opioid therapy in these conditions have not been conclusive [7-9].

There is a lack of studies on the pattern of opioid use after skeletal fractures. Most of the reports in the literature are concerned with chronic back pain or other non-cancer pain conditions, but not with skeletal trauma patients [2,10,11]. The design and results of these studies illustrate the need for more selected patient groups with specific end-point data such as skeletal injuries.

Fractures of the tibial shaft are among the most common of serious skeletal injuries [12]. They are slow to heal and frequently cause permanent sequelae [13]. We analysed the long-term pattern of opioid consumption in patients with tibial shaft fractures. We aimed to study if potential risk factors such as age, sex, type of treatment, and mechanisms of injury would predict a prolonged opioid therapy. Moreover, we wanted to assess the potential risk of dose escalations in prescribed opioids in these patients.

## Methods

Sweden has a unique personal identification number for all residents, which allows linkage between healthcare and other information from different registers for research. Data on all patients with tibial shaft fractures were obtained from the Swedish National Hospital Discharge Register (SNHDR). The Register records diagnoses and designated treatment codes according to the International Classification of Diseases (ICD), covering at least 98% of all hospital admissions in Sweden. A matched control group without tibial fractures was extracted from the Total Population Register. Each patient in the fracture group was matched with five individuals by age, sex, and residential area. None in the control group had been admitted to a hospital for a tibial fracture during the study period. Data on death or emigration for both groups were retrieved by Statistics Sweden from the Total Population Register.

Since July 1, 2005, all prescriptions filled at pharmacies in Sweden are stored in the National Pharmacy Register [14]. This does not include over-the-counter sales, which include some analgesics such as paracetamol and some of the non-steroidal anti-inflammatory drugs. However, opioid analgesics can only be obtained in pharmacies with prescription and are thereby included in the Register.

We identified all admissions in the SNHDR with ICD diagnostic codes for tibial shaft fractures (S822, S8220, and S8221). Relevant surgical intervention codes were analysed accordingly (NGJ29-NGJ99). Mechanisms of injury were studied using ICD E-codes (external codes) and grouped into 6 categories: fall on the same level, fall from height, unspecified fall, transport accident, miscellaneous, and unreported cause. The study period was July 1, 2005 to December 31, 2008.

All opioid analgesics prescribed to the patients in the study and control group were extracted from the National Pharmacy Register. These data include the following: name of the drug, date of filling the prescription, drug strength, number of pills, and dosage. The morphine equivalent dose (MED) for each opioid prescription in milligrams (mg) was calculated by multiplying the number of pills prescribed by the drug strength. These doses were then converted to MED using available equianalgesic conversions [15]. The median MED per day was calculated for each month. The MED was categorized as beeing low (< 20 mg), moderate (20–180 mg), or high (>180 mg) [16,17]. We analysed potent opioids (oxycodone, morphine, and fentanyl), whereas less potent opioids (dextropropoxyphene, codeine, and tramadol) were not included [18]. We did not want to be biased by patients with associated fractures, therefore we exluded all patients with other fractures than tibial shaft fractures. Moreover, we excluded patients who had potent opioids before the index hospitalisation, as we wanted to study new opioid use after fracture. The study was approved by the regional ethical review board located at the Karolinska Institutet (2009/837-31/3 and 2010/0125-32).

### Statistical analysis

We used descriptive statistics to define the median values with interquartile ranges (IQR). Kaplan-Meier analysis calculated the cumulative opioid consumption with 95% confidence intervals (CI). The opioid therapy was considered to be ceased when no new prescription was found during 3 consecutive months of follow-up (after 3 months a new opioid prescription has to be issued).

We used the Cox multiple-regression model to study risk factors for a prolonged opioid consumption after sustaining the fracture. Results were expressed as hazard ratios (HR) with corresponding 95% CI. If the HR is >1, the patients are more likely to end getting opioids compared with patients in the reference group. In the simple Cox model, we studied the following risk factors: age, sex, method of treatment (surgical or non-surgical) as well as mechanism of injury. All variables were later adjusted for in a multiple Cox model.

Logistic regression analysis compared the group of patients using opioids after the fracture with those who never had opioid prescriptions during follow-up. The dependent variable in the model was opioid use (yes/no) and the covariates were age, sex, type of treatment, and mechanism of injury. The level of significance was set at P < 0.05. All statistics were performed using the PASW statistics package version 18 (SPSS inc., Chicago, Illinois, USA).

## Results

### Study population

**Figure 1:**
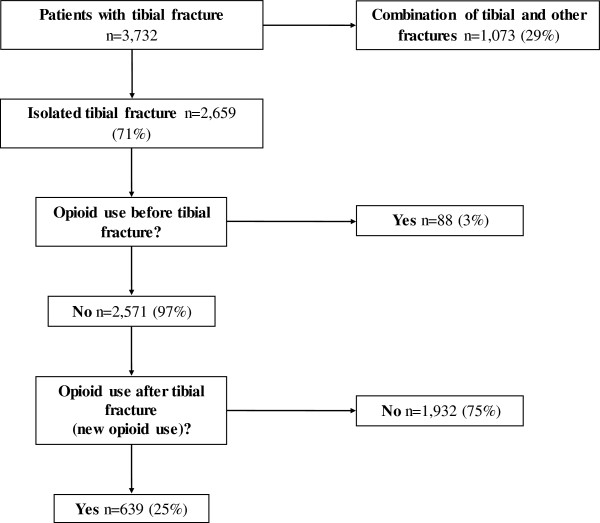
Cohort eligibility and final sample size.

We identified 3,732 patients (>= 16 years of age) who were hospitalized with tibial shaft fractures. We excluded all patients with associated fractures and patients using opioids before the index hospitalization. This left us with a final sample size of 2,571 patients. Of those, 639 (25%) had prescriptions of opioids after the fracture (new opioid use) (Figure 1). The corresponding age- and sex-matched control cohort consisted of 12,855 individuals (median age 46 [16–101] years, 62% men). Filling a prescription on opioid analgesics in the controls was seen in 353 (3%) cases during the same observation period.

Baseline data of the final study cohort with new opioid use after isolated tibial shaft fractures (n = 639) is shown in Table 1. The median age was 45 (16–97) years and most of the patients were males (61%). The type of fracture was most often a closed fracture (78%) and surgical treatment was chosen in the majority of the cases (81%). The mechanism of injury was fall on the same level in 41% of the cases, followed by transport accidents (21%). The median follow-up time after the fracture was 17 (IQR 7–27) months.

**Table 1:** Baseline characteristics

**Total number of patients**	**639**
**Age**	
Median years	45 (16–97)
**Sex**	
Male	389 (61%)
Female	250 (39%)
**Type of fracture**	
Closed	499 (78%)
Open	103 (16%)
Unspecified	37 (6%)
**Treatment**	
Surgical	520 (81%)
Non-surgical	119 (19%)
**Mechanisms of injury**	
Fall on the same level	262 (41%)
Fall from height	66 (10%)
Fall unspecified	53 (8%)
Transport accident	133 (21%)
Miscellaneous	115 (18%)
Missing	10 (2%)

### Opioid prescriptions

**Figure 2:**
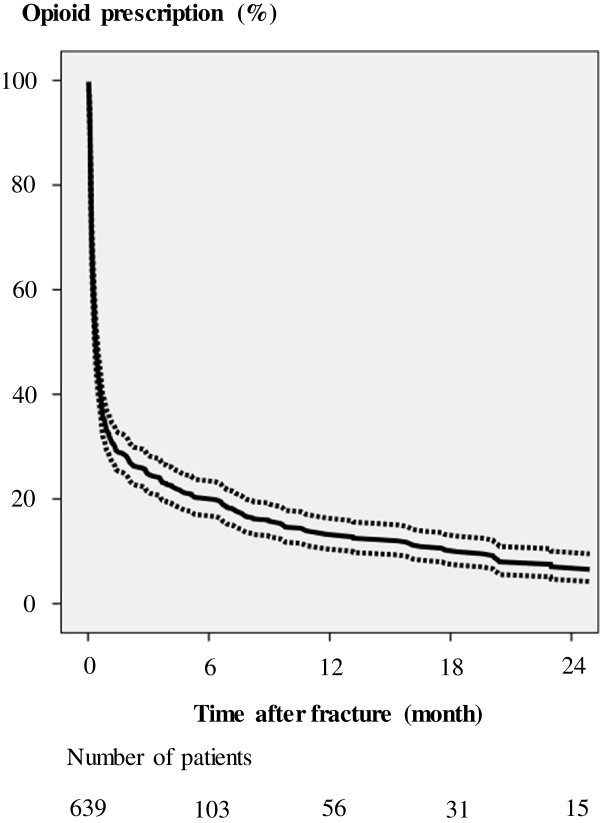
Kaplan-Meier-analysis with 95% confidence intervals of the last opioid prescription of patients with tibial fractures.

**Figure 3:**
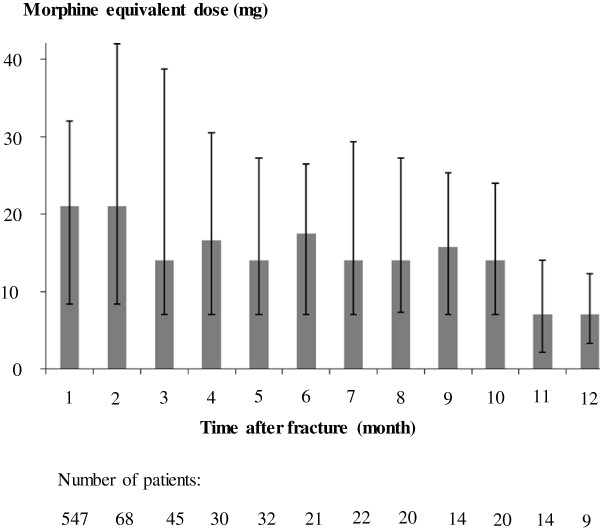
The median (interquartile range) morphine equivalent dose (MED) in milligram (mg) per day prescribed to patients with tibial fractures in different time periods after fracture.

Kaplan-Meier analysis revealed that 6, 12, and 18 months after sustaining the tibial fracture, 21% (95% CI 17–24), 14% (11–17), and 11% (8–13) still required opioid prescriptions, respectively (Figure 2). The median daily MED was 21 (IQR 8–32) mg within the first month after the fractures for those patients who were started on opioids (Figure 3).

**Figure 4:**
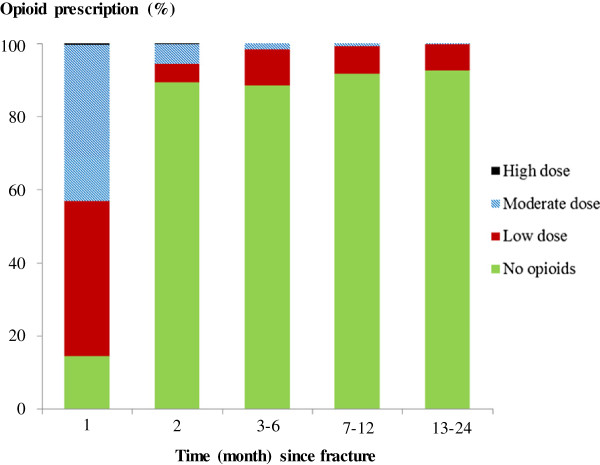
The distribution of opioid prescriptions in 639 patients in different time intervalls after tibial fracture (low dose, < 20 mg MED per day; moderate, 20 < 180 mg; high > 180 mg; MED = morphine equivalent dose).

Figure 4 shows the distribution of patients on various doses during different exposure windows. The first prescription of opioids was filled during the first month after fracture by the majority of the patients (86%) (Figure 4). During the study period, the proportion of patients using moderate and high doses decreased and the proportion of patients who stopped taking opioid drugs increased (Figure 4).

Comparison of the daily MED among individuals who both had prescriptions during the first 3 months and the 6th month indicated that the majority of these patients (11/14) did not have dose escalations (an increase by more than 30% of the original dose).

The simple (unadjusted) Cox regression-analysis showed that older patients (> 50 years) (HR 1.7), women (HR 1.3), and non-surgical treatment (HR 1.4) made it less likely to continue opioid analgesic use. After adjustment for covariates in the multiple Cox analysis, older age was still a statistically significantly associated with ending opioid use sooner (HR 1.5) (Table 2).

**Table 2:** 639 patients with new opioid use after tibial fracture (Cox regression analysis)

			**Simple Cox regression**^**a**^			**Multiple Cox regression**^**b**^		
**Covariate**		**No. of patients**	**HR**	**95% CI**	**p-value**	**HR**	**95% CI**	**p-value**
**Age**	< 50 years	364	Reference			Reference		
	> 50 years	275	1.7	1.4-2.0	0.001	1.5	1.3-1.9	0.001
**Sex**	Male	389	Reference			Reference		
	Female	250	1.3	1.1-1.6	0.002	1.1	0.9-1.4	0.2
**Type of fracture**	Closed	499	Reference			Reference		
	Open	103	0.9	0.7-1.2	0.4	0.9	0.7-1.1	0.3
	Unspecified	37	0.6	0.4-0.8	0.004	0.6	0.4-0.9	0.022
**Treatment**	Surgical	520	Reference			Reference		
	Non-surgical	119	1.4	1.1-1.8	0.004	1.1	0.9-1.4	0.6
**Mechanism of injury**	Fall on the same level	262	Reference			Reference		
	Fall from height	66	0.9	0.6-1.2	0.4	0.8	0.6-1.1	0.2
	Fall unspecified	53	0.9	0.6-1.2	0.5	0.9	0.6-1.2	0.5
	Transport accident	133	1.1	0.9-1.4	0.4	0.9	0.7-1.2	0.5
	Miscellaneous	115	1.2	1.0-1.6	0.1	1.1	0.8-1.4	0.6
	Missing	10	1.0	0.6-2.0	0.9	1.0	0.5-1.8	0.9

HR = hazard ratio, CI = confidence interval, ^a^crude, ^b^adjusted for age, sex, type of fracture, treatment, and mechanism of injury; if the HR is >1, the patients are more likely to end opioid intake compared with the reference group.

Patients with isolated tibial fractures who received opioids after fracture (n = 639) were compared with the patients who did not get opioid prescriptions (n = 1,932). There was no difference concerning age, sex, and mechanism of injury between the 2 groups (data not included). However, patients receiving opioids during follow-up were more likely to have undergone surgery for the fracture (odds ratio 2.3, 95% CI 1.7-2.6, p < 0.001).

## Discussion

There has been a continuous increase of opioid use for pain treatment among patients with non-cancer pain conditions during the past decade [19]. We studied the long-term opioid prescriptions after tibial shaft fractures in a national Swedish study. 25% of the patients filled a prescription for opioid analgesics at some point after the fracture. However, the doses prescribed were rather low and we did not see any evidence of a major dose escalations over time.

We excluded all patients with potent opioid prescriptions prior to the fracture, as we wanted to study the occurrence of new opioid prescriptions. Moreover, we excluded patients with other fracture diagnoses as we wanted study a rather homogenous fracture cohort. We are aware that the included patients may have obtained opioids during follow-up due to other reasons than the skeletal injury such as back pain, extremity pain, and abdominal pain. Therefore, an age- and sex-matched control cohort without fracture was included for comparison. In a cross-sectional survey from 2010 based on a nationwide register in Denmark, a high overall prevalence of opioid consumption (4.5%) was found in the general population. The relevance of which was, however, unknown [20]. These findings are in accordance with our findings of an opioid use of 3% in the control cohort without tibial fracture.

We did not detect any indication of major dose escalation in our cohort during the follow-up period. The median daily MED was between 7 and 21 mg during month 1 and 12 after fracture. Furthermore, as shown in Figure 4, the median MED for patients taking opioids was predominantly moderate to low in the beginning. During follow-up, the frequency of patients on moderate and high doses reduced. This is consistent with other data concerning non-trauma related pain conditions. In a meta-analysis of efficacy and safety of long-term opioid therapy for chronic non-cancer pain, many patients discontinued the therapy and very few patients showed signs of opioid addiction or abuse [21].

Our finding of a higher risk for continued use of opioids in younger patients (< 50 years) may be explained by a more extensive injuries often sustained during transport accidents in comparison with falls on the same level which is more often seen in elderly people. Furthermore, this finding for the older patient group may be reassuring, recently published reports raised increasing concerns on the safety of opioid analgesics in elderly people [22-24].

The shortcomings of our study include the following: opioids prescribed to patients is not always equivalent to requirement or consumption of opioids. The incidence figures in this study may present and over- or underestimation of actual opioid use. An overestimation of the use of potent opioid analgesics is due to the fact that not all prescribed drugs are consumed. Thus the actual number of consumed doses is probably lower than 100 percent. In contrast, we did not include less potent opioids such as codein which converts to morphine in the liver, resulting in an underestimation. Moreover, we did not analyse other analgesics such as COX-inhibitors which may augment the analgesic effect of opioids reducing the quantity of the consumed opioid required. A further limitation of the study is: we do not know anything about the efficacy of the analgesic treatment. Lack of analgesic effect and/or side effects of opioids are major reasons why opioid therapy is stopped [25]. Patients may also, after some time, be prescribed less potent opioids by their general practitioners, who may be reluctant to provide potent opioids for non-cancer pain. This is a register study, therefore we do not know the specific reason why the patients discontinued the use of opioid medication. For example, one reason for discontinuing opioid treatment for elderly patients could be due to a higher incidence of adverse events. We also do not know if the excluded patients, who were already taking opioids prior to their tibial fracture, had an increase in their prescribed opioids following the fracture.

Our study is based on well validated government controlled national registries, including all hospitalized patients and opioid prescriptions in Sweden. We only studied the use of strong opioids in order to get a more homogenous patient group and to guarantee that the patients’ consumption is registered in order to obtain accurate statistics regarding drug escalation problems.

## Conclusions

We did not see any signs from registry-data of major dose escalations over time among patients who received prescriptions for potent opioid analgesics after tibial shaft fractures.

## Competing interests

The authors declare that they have no competing interests.

## Authors’ contributions

ZAD and RJW: planning, data analysis, statistics, writing, and editing of the manuscript. KÅJ, COS, and SM: planning, writing, and editing of the manuscript. All authors read and approved the final manuscript.

## Pre-publication history

The pre-publication history for this paper can be accessed here:

http://www.biomedcentral.com/1471-2253/14/4/prepub
